# Harmonization of summaries of product characteristics (SmPCs) of drugs with the same active ingredients: an evaluation of SmPCs of the most frequently prescribed active substances

**DOI:** 10.1007/s00228-021-03209-7

**Published:** 2021-10-27

**Authors:** Maximilian Gahr, Bernhard J. Connemann, Rainer Muche, René Zeiss, Almuth Wolf

**Affiliations:** 1 Department of Psychiatry and Psychotherapy III, University Hospital of Ulm, Leimgrubenweg 12-14, 89075 Ulm, Germany; 2 Institute of Epidemiology and Medical Biometry, University of Ulm, Schwabstr. 13, 89075 Ulm, Germany

**Keywords:** Aut-idem, Generic drug, Generic substitution, Package insert, Pharmacovigilance, Prescribing information

## Abstract

**Purpose:**

In aut-idem or generic substitution, discrepancies between summaries of product characteristics (SmPCs) referring to the same active substance (AS) may cause difficulties regarding informed consent and medical liability. The qualitative and quantitative characteristics of such discrepancies are insufficiently studied, impeding harmonization of same-substance SmPCs and compromising safe drug treatment.

**Methods:**

SmPCs of the one hundred most frequently prescribed ASs in Germany were analyzed for discrepancies in the presentation of indications (Inds) and contraindications (CInds). Inclusion and exclusion criteria of drugs/SmPCs were chosen according to the standards of the aut-idem substitution in Germany.

**Results:**

According to the study protocol, we identified 1486 drugs, of which 1426 SmPCs could be obtained. 41% respectively 65% of the ASs had same-substance SmPCs that differed from the respective reference SmPC in the number of listed Inds respectively CInds. The number of listed Inds/CInds varied considerably between same-substance SmPCs with maximum ranges in Inds of 7 in amoxicillin, and in CInds of 11 in lisinopril. Many ASs had large proportions (> 50%) of associated same-substance SmPCs that differed from the respective reference SmPC. A considerable proportion of ASs had same-substance SmPCs with formal and content-related differences other than the discrepancy in the number of Inds/CInds.

**Conclusion:**

This evaluation of same-substance SmPCs shows a clear lack of harmonization of same-substance SmPCs. Considering that generic substitution has become the rule and that physicians usually do not know which drug the patient receives in the pharmacy, these discrepancies raise several questions, that require a separate legal evaluation.

## Introduction

For economic reasons, the supply with generic medication is politically preferred in most countries. In the European Union, different national regulatory frameworks exist to ensure the preferential prescription and/or the dispensing of generic drugs. In Germany, the so-called "aut-idem substitution" which is the term for the legal, regulatory and operative framework concerning generic substitution in Germany obliges pharmacists to dispense a same-substance medication of the lower third of the price range, if the respective drug is prescribed at the expense of the statutory health insurance and the responsible physician does not explicitly exclude the substitution of the prescribed product by a generic drug [1]. In Germany, exclusion of the aut-idem or generic substitution constitutes an additional expense and, in addition, requires an individual justification by the physician (which is usually not accepted by the health insurance companies). Therefore, the aut-idem or generic substitution has become the rule in Germany [2]. However, reduced efficacy and tolerability and differences in bioequivalence, particularly regarding psychoactive and antiepileptic drugs were found in association with switching from brand-name to generic medication (resp. aut-idem substitution) and vice versa and/or switching generic drugs [3–9]. Moreover, discrepancies between SmPCs of drugs with the same active ingredients were found in several studies [10–13], although regulations of the European Medicines Agency (EMA) provide that the content of summaries of product characteristics (SmPCs, which are the European counterpart of the US prescribing information) of generic drugs "… should be in all relevant aspects consistent with that of the reference medicinal product except for indications or dosage form still covered by patent law…" [14] and the EMA and the Committee for Medicinal Products for Human Use (CHMP) are working with harmonization of SmPCs (by post-authorisation referral procedures [15] and guidelines [16]). These discrepancies between same-substance SmPCs may refer to *actual differences* between the corresponding same-substance products (regarding regulatory aspects as approved indications, but also pharmacologic aspect as excipients, additives, and carriers of the active substance, available strengths, dosage form, appearance and size of the drug, bioavailability, galenical properties, etc.) and/or may be *self-referential* in terms of discrepancies in form and content of SmPCs without clear reference to possible regulatory and/or pharmacologic/galenic differences between the corresponding same-substance products. Since the physician usually does not know, which generic drug is dispensed in the pharmacy, the differences between same-substance drugs and the discrepancies between same-substance SmPCs may cause difficulties regarding informed consent and may have implications for medical liability. Qualitative and quantitative characteristics of discrepancies between same-substance SmPCs are not sufficiently known, which impedes harmonization of same-substance SmPCs and compromises safe drug treatment. Therefore, we have analyzed selected paragraphs of the SmPCs of the most frequently prescribed active substances in Germany regarding type and extent of discrepancies between same-substance SmPCs.

### Ethical approval

No personal data were used in the present study. According to the guidelines and regulations of the local ethics committee (Ulm University) no ethical approval is necessary in such cases.

### Period of data collection

The data were collected from August 3, 2020, until October 27, 2020. Data regarding over-the-counter drugs were collected from January 26, 2021, until February 28, 2021.

### Selection of active substances

The one hundred most frequently prescribed active substances in Germany in 2018 were included and ranked according to the number of annual prescriptions.^1^ Active substances and quantities of annual prescriptions were identified by the "Arzneiverordnungs-Report 2019" (AVR) [2]. The AVR is an annual scientific document published since the year 1985 and contains data, costs, and analyses of drug prescriptions of the previous year, prescribed at the expense of the statutory health-insurance companies in Germany. Over-the-counter drugs that are prescribed at the expense of the statutory health-insurance companies in Germany are also considered in the AVR.

Excluded active substances (see next paragraph) were replaced by subsequent active substances, and the list of active substances was extended accordingly by adding the 101st, 102nd ranked active substance, etc., until the list contained one hundred active substances.

### Inclusion and exclusion criteria of drugs respectively SmPCs

As every SmPC constantly refers to a specific medicinal product and not to an active substance, the inclusion and exclusion criteria, which are explained in the following, always refer to an individual pair of a drug and the corresponding SmPC.

Inclusion and exclusion criteria of drugs or SmPCs were chosen according to the standards of the aut-idem substitution in Germany: This implies that (1) (according to part B of the annex VII to paragraph M of the drug directive of the Federal Joint Committee (G-BA), which constitutes the so-called "Substitutionsausschlussliste") [17]) specific active substances and/or combinations of active substances are a priori explicitly excluded from the generic substitution (in our study levothyroxine sodium and the combination of levothyroxine and potassium iodate were excluded), and (2) only drugs that are identical to the prescribed drug regarding strength and package size, (3) only drugs that are approved for the treatment of at least one similar disorder, and (4) only drugs that feature the same or an exchangeable dosage form may be dispensed as aut-idem substitutes in the pharmacy. In order to exclude "real" drug-related differences as reasons for discrepancies between same-substance SmPCs, only same-substance products with identical strength, package size and dosage form were included. Therefore, the most frequently prescribed strength of an active substance or product was identified by the AVR and used for further data acquisition. To further harmonize the characteristics of the included SmPCs, same-substance SmPCs referring to the most frequently prescribed package size of an active substance (identified by the AVR) were included. There are three normed package sizes in Germany: N1 (small package size: 16–24 units), N2 (moderate: 45–55 units), and N3 (large: 95–100 units). Apart from seven active substances/ingredient combinations (azithromycin, colecalciferol, dexamethasone/gentamicin, fosfomycin, ivy leaves, paracetamol and xylometazoline) (here, the most frequently prescribed package size was N1) the most frequently prescribed package size was N2. Finally, based on the data obtained via the German online portal for drug information of the federal and state government ("PharmNet.Bund"; for further details see next paragraph)^2^ the dosage form with the most same-substance drugs was selected (in most active substances oral dosage forms) and used for further data acquisition. Only products that were marketable and approved in Germany during the period of the data collection were included. Parallel imported and reimported drugs were excluded as these products are subject to a simplified or—in cases of Europe-wide marketing authorisation—no marketing authorisation procedure, which implies that SmPCs of those products are merely translations of the SmPC of the original product. Products, which are manufactured according to officially normed standard formulations (so-called "Standardzulassungen") and therefore exempt from the marketing authorisation procedure were also excluded. (These products are predominantly only available in pharmacies as store brands, and their SmPCs were frequently unavailable). Homeopathic medicinal products were excluded. Finally, if only one same-substance product was identified according to the mentioned inclusion and exclusion criteria, the respective active substance (ambroxol, physiological saline solution, noscapine, iron (II) glycine sulfate, imidazole/triazole combined with corticosteroids) was excluded.

### Identification of products and acquisition of SmPCs

The German online portal for drug information of the federal and state government^3^ ("PharmNet.Bund") was used for identification of the included products by using the name of the active substances identified by the AVR and the corresponding code of the Anatomical Therapeutic Chemical (ATC) Classification System [18]. The portal makes drug-related data of the higher German federal authorities centrally and transparently available to the public. In addition, it provides applications for communication, authorisation, and registration of drug-related data to public authorities and pharmaceutical manufacturers. Furthermore, an integrated online database and search tool ("Arzneimittel-Informationssystem" [AMIS]^4^) provides drug information as name of the drug, dosage form, authorisation holder and number, information regarding marketability, SmPC and patient package insert, etc. free of charge. The content of this database is maintained and daily updated by the Federal Institute for Drugs and Medical Devices, the Paul Ehrlich Institute, and the Federal Office of Consumer Protection and Food Safety. Following identification of included products by AMIS according to the above-mentioned inclusion and exclusion criteria, the corresponding SmPCs were obtained directly in AMIS or by using the online portals "Rote Liste"^5^ and/or "Gelbe Liste".^6^ If the SmPCs were not available by searching one of these online portals, the respective SmPC was requested directly by the manufacturer by email.^7^ In order to exclude discrepancies between same-substance SmPCs due to differing update status of SmPCs, all same-substance SmPCs were obtained, respectively requested within one day.

### Analysis of SmPCs and examined parameters—primary analysis (number of Inds and CInds)

The number of indications (Ind) and contraindications (CInd) were analyzed. Therefore, the standardized paragraphs of the SmPCs "4.1 Indications" and "4.3 Contraindications" of each included SmPC were investigated and the number of listed Inds and CInds was counted and documented. In the *primary evaluation* of the Inds and CInds apparent discrepancies in the number of Inds and CInds due to formal discrepancies between same-substance SmPCs in the presentation of Inds and CInds were not considered. Therefore, the *primary evaluation* strictly focused the content of the respective paragraph and disregarded formal discrepancies. Discrepancies in content were decisive for the identification of discrepancies in the number of Inds and CInds and discrepancies in formal aspects were not considered in this part of the analysis.^8^ In principle, only absent or additional Inds/CInds in the respective paragraph led to identification of discrepancies in the number of Inds/CInds. For every active substance, the reference SmPC for the identification of absent or additional Inds/CInds was the most frequent type of a same-substance SmPC. (The reference SmPC thus represents the largest group of harmonized same-substance SmPCs relating to all SmPCs referring to the same active substance.)

Groups of SmPCs referring to the same active substance were created and the associated numbers of Inds and CInds were compared. To describe the degree of harmonization of a group of same-substance SmPCs the range (= difference between maximum and minimum value) of the number of Inds and CInds of the associated same-substance SmPCs was calculated. In addition, the proportion of same-substance SmPCs with differing numbers of Inds/CInds was calculated by the number of discrepant SmPCs divided by the total number of SmPCs referring to the respective active substance. For every active substance, the reference SmPC for the qualitative and quantitative evaluation of the Inds/CInds was the most frequent type of a same-substance SmPC.

### Analysis of SmPCs and examined parameters—secondary analysis (other aspects)

In the *secondary analysis*, other discrepancies between same-substance SmPCs, e.g., regarding formal and/or content-related aspects, were analyzed. The reference SmPC for the identification of such discrepancies was the most frequent type of a same-substance SmPC respectively the SmPC that represented the majority of harmonized same-substance SmPCs. We defined five types of discrepancies. These are elucidated in Table 1.

### Statistical analysis

All analyses were performed with Microsoft Excel (version 2019).

## Results

### Basic aspects

According to the study protocol we identified 1486 drugs (referring to 100 active substances), of which 1426 SmPCs could be obtained. Sixty SmPCs could not be obtained with any of the mentioned data acquisitions methods. Seven active substances/combinations of active substances had to be excluded according to the inclusion and exclusion criteria.^9^ The most frequently prescribed dosage forms of the identified 100 most frequently prescribed active substances were oral (film-coated tablet: 34%; tablet: 25%; hard capsule: 7%; sustained-release tablet: 5%; powder/granulate for oral solution: 3%; oral drops: 2%; oral solution: 1%; soft capsule: 1%), followed by powder/suspension for inhalation (7%), solutions for injection (5%), eye drops (3%), cream (3%), nasal drops (2%), suppositories (1%), and transdermal patches (1%). Table 2 demonstrates the included active substances, the associated annual prescriptions, dosage forms, and strengths.

### Discrepancies in the number of Inds (primary analysis)

Discrepancies between same-substance SmPCs in the number of listed Inds were found in 41 (41%) of included active substances. The largest ranges between minimum and maximum number of Inds of same-substance SmPCs were found in amoxicillin (range = 7), ibuprofen (range = 5), and allopurinol, clopidogrel, ramipril, escitalopram, sertraline, and sulfamethoxazole/trimethoprim (in each case range = 4). The largest proportion of SmPCs with a divergent number of Inds were found in sulfamethoxazole/trimethoprim (62.5%),^10^ mometasone (50%), formoterol (50%), bisoprolol (47.4%), valproic acid (46.7%), macrogol (46.2%), levodopa/benserazide (42.9%), hydrochlorothiazide (38.9%), betamethasone (37.5%), citalopram (36%), codeine (33.3%), amitriptyline (25%), ciprofloxacin (23.5%), amoxicillin, budesonide, and salbutamol (in each case 20%). Table 3 demonstrates number of evaluated same-substance SmPCs, number of listed Inds (and mean, median, maximum and minimum number of Inds, range, and standard deviation), and absolute and relative frequencies of drugs/SmPCs with differing numbers of Inds.

### Other formal and content-related discrepancies in the presentation of Inds (secondary analysis)

A discrepancy of type A was found in one SmPC (1%) (referring to the ingredient combination levodopa/benserazide), and a discrepancy of type B was found one in each SmPC of the following three substances: amoxicillin, amoxicillin/clavulanic acid, and sulfamethoxazole/trimethoprim (3%). A discrepancy of type C and D was not found in the presentation of Inds (see Table 4).

### Discrepancies in the number of CInds (primary analysis)

Discrepancies between same-substance SmPCs regarding the number of listed CInds were found in 65 (65%) of included active substances. The largest ranges between minimum and maximum number of CInds of same-substance SmPCs were found in lisinopril (range = 11), hydrochlorothiazide (range = 9), torasemide (range = 8), carvedilol (range = 7), betamethasone, formoterol, lorazepam, metoprolol, oxycodone (each range = 6), and mometasone (range = 4). The largest proportions of same-substance SmPCs with a divergent number of CInds were found in carvedilol (71.4%), torasemide (61.5%), mometasone (66.7%), betamethasone (62.5%), xylometazoline (59.3%), metoclopramide (57.4%), metformin (53.8%), cholecalciferol, doxycycline, estriol, formoterol, lorazepam, sulfamethoxazole/trimethoprim (each 50%), bisoprolol (47.4%), oxycodone (46.4%), lercanidipine, metoprolol, pantoprazole, penicillin (in each case 44.4%), ibuprofen (42.9%), and human insulin (40%).

Table 5 demonstrates number of evaluated same-substance SmPCs, number of listed Inds (and mean, median, maximum and minimum number of Inds, standard deviation), and absolute and relative frequencies of drugs/SmPCs with discrepancies in the number of CInds.

### Other formal and content-related discrepancies in the presentation of CInds (secondary analysis)

A discrepancy of type A was found in 25 SmPCs referring to 13 active substances (13%), type B in five SmPCs referring to four active substances (4%), and type C in 40 SmPCs referring to 13 active substances (13%). Twenty-seven SmPCs referring to ten active substances (10%) featured a discrepancy of type D. Two types of discrepancies in one SmPC (combined type) were found in eight active substances (8%) referring to 17 SmPCs. For details see Table 6.

## Discussion

In the present study, the SmPCs of the one hundred most frequently prescribed active substances in Germany in the year 2018 were analyzed for discrepancies in the presentation of Inds and CInds. We found considerable proportions of included active substances with differing same-substance SmPCs: 41% respectively 65% of the active substances had same-substance SmPCs that differed in the number of listed Inds respectively CInds from the reference SmPC. In addition, we found many active substances with remarkably large proportions of same-substance SmPCs (> 50%) that diverged from the respective reference SmPC, particularly in the number of CInds. The number of listed Inds/CInds varied considerably between same-substance SmPCs with maximum ranges regarding Inds of 7 in amoxicillin, and CInds of 11 in lisinopril. Other discrepancies which refer to the wording and/or position of the respective Inds/CInds in the SmPC were also analyzed in order to contextualize the primary findings of discrepancies in the mere number of Inds and CInds. Here, we also found considerable proportions of active substances that featured same-substance SmPCs with formal and content-related differences other than the discrepancy in the number of Inds and CInds. To summarize, this evaluation of same-substance SmPCs demonstrates a clear lack of harmonization of same-substance SmPCs.

Taken into account that generic substitution has become the rule [2] and the physician usually does not know which drug the patient receives in the pharmacy, the discrepancies between same-substance SmPCs found in our study raise several questions, particularly regarding informed consent and medical liability. Indeed, the standards of the aut-idem substitution in Germany are pursuing safe drug treatment in generic substitution by ensuring the substitution of only nearly "identical products". Nevertheless, differences between substitutable same-substance drugs in regulatory aspects and/or pharmacologic/galenic properties (e.g., approved indications, excipients, additives, carriers of the drug, dosage form, appearance and size of the drug, bioavailability etc.) are permitted. It should be considered that switching from brand-name to generic medication may be associated with reduced efficacy and tolerability, and differences in bioequivalence, particularly regarding psychoactive drugs, as shown in several studies [3–9]. Given the large number of same-substance drugs that can be mutually exchanged in the pharmacy and the volatility of the generic drug market, the physician is virtually incapable of being sufficiently aware of the discrepancies between same-substance drugs and/or SmPCs. How can the physician then attend to his duty of informed consent? Who is legally responsible in the case of damage due to adverse drug reactions or insufficient therap response that could have been avoided in the case of adequate information and individualized selection of a product? These aspects require a separate legal evaluation. Moreover, regulatory efforts appear necessary in order to improve harmonization of same-substance SmPCs.

## Limitations

The extent of the analyzed data did not allow a content-related evaluation of the identified discrepancies. Therefore, we cannot determine, if the discrepancies found in our study correspond to real differences between the included same-substance drugs in regulatory aspects (e.g., different approved indications) and/or pharmacologic/galenic properties or merely represent errors in particular SmPCs (e.g., insufficient updating, production error). In this respect it has to be considered that the clinical relevance of the discrepancies found in our study remains unclear and requires. An adequate evaluation of the clinical relevance of these discrepancies requires a thorough analysis of contents and qualitative aspects of the diverging same-substance SmPCs. Furthermore, it is reasonable to assume that discrepancies between same-substance SmPCs are more common and of greater clinical importance for drugs approved by the national procedure, or by mutual recognition, than by the central procedure. Since we did not collect data on the type of approval procedure and the year of first approval of the included drugs, we are not able to provide evidence for this assumption. In addition, considering the volatility of the generic drug market and that SmPCs are subject to continuous adjustment, our results exclusively represent the SmPC-situation during the period of the data collection. Finally, we have evaluated SmPCs of drugs approved in Germany. Taken into account country-dependent differences in regulatory frameworks and drug markets, our results cannot be easily transferred to other countries. Therefore, it is unclear if the quantitative and qualitative discrepancies of same-substance SmPCs demonstrated by our analysis can be found in other countries.

## Conclusions

There are considerable discrepancies in the number of listed indications and contraindications between SmPCs of drugs with the same active ingredient. Further studies should address content-related aspects of these discrepancies. Nevertheless, our findings raise questions regarding informed consent and medical liability, which require a separate legal evaluation. Regulatory efforts appear necessary to improve the harmonization of same-substance SmPCs.

**Table 1:** Definition of types of discrepancies between same-substance SmPCs other than differences in the number of Inds and CInds

Type of discrepancy	Definition of the type of discrepancy
Type A	According to the evaluation protocol no discrepancies in the number of Inds and CInds, but content-related differences were found (e.g. an Ind/CInd of the reference SmPC is replaced by a different Ind/CInd in one or more SmPCs referring to the same substance).
Type B	Discrepancy in number and content of Inds/CInds was found, e.g., some Inds/CInds of the reference SmPC are not listed and replaced by more and different Inds/CInds. The quantitative extent of the overall discrepancy between the respective same-substance SmPCs is therefore underestimated by only using the range.
Type C	Discrepancy in the number of CInds was found. (This type of discrepancy was found only in the presentation of CInds.) The respective CInds are not listed in the respective paragraph (4.3), however are shown in another paragraph of the SmPC (e.g. 4.4 or 4.6) completely (type C^1^) or incompletely (type C^2^), and with identical (type C^a^) or different content (type C^b^). Type C^a+b^ was used to describe SmPCs with > 1 CInds absent in paragraph 4.3 and listing of some absent CInds in identical and some CInds in different wording in another paragraph.
Type D	Discrepancy in the number of CInds was found. (This type of discrepancy was found only in the presentation of CInds.) This discrepancy is due to the following: CInds that are listed in paragraphs other than 4.3 in all same-substance SmPCs are additionally listed in paragraph 4.3 in some SmPCs. (This discrepancy is due to the additional listing of CInds in paragraph 4.3 in some SmPCs, whereby these CInds are listed in all same-substance SmPCs in a paragraph other than 4.3.)
Combined type	Two types of discrepancies (A, B, C or D) are found in one SmPC. This is expressed as "type x+y".

*Ind* indication, *CInd* contraindication, *SmPC* summary of product characteristics

**Table 2:** Included substances, associated annual prescriptions in the year 2018, dosage forms, and strengths^a^

Substance/ingredient combination	Annual prescriptions (x 10^6^)	Dosage form	Strength
Ibuprofen	26.2	Film-coated tablet	600 mg
Metamizole	25.6	Oral drops	500 mg/ml
Pantoprazole	21.1	Gastro-resistant (enteric-coated) tablet	40 mg
Ramipril	20.2	Tablet	5 mg
Bisoprolol	16.9	Film-coated tablet	5 mg
Metoprolol	16.8	Sustained-release tablet	47,5 mg
Amlodipin	13.4	Tablet	5 mg
Simvastatin	12.5	Film-coated tablet	20 mg
Torasemide	11.2	Tablet	10 mg
Metformin	9.2	Film-coated tablet	1000 mg
Candesartan	8.1	Tablet	16 mg
Acetyl salicilic acid	7.0	Tablet	100 mg
Atorvastatin	6.9	Film-coated tablet	40 mg
Diclofenac	6.6	Hard capsule	75 mg
Salbutamol	6.5	Compressed gas inhalation / suspension	0,12 mg/puff
Omeprazole	6.2	Gastro-resistant (enteric-coated) hard capsule	20 mg
Allopurinol	6.1	Tablet	300 mg
Prednisolone	6.0	Tablet	5 mg
Amoxicillin	6.0	Film-coated tablet	500 mg
Xylometazoline	5.6	Nasal drops / spray	1 mg / ml
Tilidine / naloxone	5.4	Sustanied-release tablet	50 mg / 4 mg
Valsartan	4.6	Film-coated tablet	160 mg
Ramipril / hydrochlorothiazide	4.4	Tablet	5 mg / 25 mg
Cefuroxime	4.1	Film-coated tablet	500 mg
Hydrochlorothiazide	4.0	Tablet	25 mg
Pregabalin	3.9	Hard capsule	75 mg
Tamsulosin	3.8	Hard capsule	0,4 mg
Lecarnidipine	3.4	Film-coated tablet	10 mg
Apixaban	3.2	Film-coated tablet	5 mg
Paracetamol	3.2	Suppositories	75 mg
Enalapril	3.2	Tablet	10 mg
Insulin glargine	3.1	Solution for injection (pre-mixed pen)	300 IU
Mirtazapine	3.1	Film-coated tablet	15 mg
Candesartan / hydrochlorothiazide	3.1	Tablet	16 mg / 12,5 mg
Colecalciferol	3.1	Soft capsule	20 mg
Citalopram	2.8	Film-coated tablet	20 mg
Ciprofloxacin	2.8	Film-coated tablet	500 mg
Azithromycin	2.8	Film-coated tablet	500 mg
Tramadol	2.8	Sustained-release tablet	100 mg
Levodopa / benserazide	2.7	Tablet	100 mg / 25 mg
Rivaroxaban	2.6	Film-coated tablet	20 mg
Formoterol / Beclomethasone	2.6	Compressed gas inhalation, solution	6 µg / 100 µg
Quetiapin	2.5	Film-coated tablet	25 mg
Phenprocoumon	2.4	Tablet	3 mg
Sitagliptin	2.4	Film-coated tablet	100 mg
Furosemide	2.4	Tablet	40 mg
Moxonidine	2.3	Film-coated tablet	0,2 mg
Zopiclone	2.3	Film-coated tablet	7,5 mg
Metoclopramide	2.3	Tablet	10 mg
Venlafaxine	2.2	Sustained-release hard capsule	75 mg
Enoxaparin	2.2	Soution for injection	4000 IU
Spironolactone	2.2	Tablet	50 mg
Valsartan / hydrochlorothiazide	2.1	Film-coated tablet	160 mg / 12,5 mg
Opipramol	2.1	Film-coated tablet	50 mg
Amoxicillin / clavulanic acid	2.0	Film-coated tablet	875 mg / 125 mg
Lorazepam	1.9	Tablet	1 mg
Nebivolol	1.9	Tablet	5 mg
Metformin / sitagliptin	1.9	Film-coated tablet	1000 mg / 50 mg
Amitriptyline	1.9	Film-coated tablet	25 mg
Dexamethasone / gentamicin	1.9	Eye drops	1 mg / 5 mg
Doxycycline	1.9	Tablet	100 mg
Estriol	1.9	Vaginal creme	1 mg
Clindamycin	1.9	Film-coated tablet	600 mg
Etoricoxib	1.9	Tablet	90 mg
Lisinopril	1.9	Tablet	10 mg
Ofloxacin	1.9	Eye drops	3 mg
Risperidone	1.8	Film-coated tablet	0,5 mg
Macrogol	1.8	Powder for oral solution	13,125 g
Fentanyl	1.8	Transdermal patch	5,78 mg (25 µg / h)
Fosfomycin	1.8	Granulate for oral solution	3000 mg
Phenoxymethylpenicillin	1.8	Film-coated tablet	980,4 mg
Gabapentin	1.8	Hard capsule	300 mg
Mometasone	1.7	Nasal drops / spray	0,052 mg (50 µg / spray)
Clopidogrel	1.7	Film-coated tablet	75 mg
Ivy leaves	1.7	Oral solution	7 mg / ml
Human insulin	1.7	Solution for injection	300 IU
Budesonide	1.7	Powder for inhaltion	0,2 mg
Salmeterol / fluticasone	1.7	Powder for inhalation	50 µg / 500 µg
Carvedilol	1.7	Tablet	25
Methylphenidate	1.7	Hard capsule	20
Insulin lispro	1.6	Solution for injection	300 IU
Insulin aspart	1.6	Solution for injection	300 IU
Tiotropium bromide	1.6	Hard capsule with powder for inhalation	18 µg
Formoterol / budesonide	1.6	Powder for inhalation	9 µg / 320 µg
Methocarbamol	1.6	Film-coated tablet	750 mg
Melperone	1.5	Film-coated tablet	25 mg
Betamethasone	1.5	Creme	1,214 mg
Esomeprazole	1.5	Gastro-resistant (enteric-coated) tablet	40 mg
Latanoprost	1.5	Eye drops	0,05 mg
Levetiracetam	1.5	Film-coated tablet	500 mg
Sulfamethoxazole / trimethoprim	1.5	Tablet	800 mg / 160 mg
Codeine	1.4	Tablet	30 mg
Sertraline	1.4	Film-coated tablet	50 mg
Escitalopram	1.4	Film-coated tablet	10 mg
Mometasone	1.4	Creme	1 mg
Formoterol	1.3	Hard capsule with powder for inhalation	12,5 µg
Oxycodone	1.3	Sustained-release tablet	10 mg
Zolpidem	1.3	Film-coated tablet	10 mg
Valproic acid	1.3	Sustained-release tablet	300 mg
Cefaclor	1.3	Granulate for oral solution	250 mg

*IU* international unit

^a^presented in descending order by annual prescriptions referring to the year 2018 (~ number of annual prescriptions of any of the available package sizes of a drug containing the respective substance)

**Table 3:** Discrepancies between same-substance SmPCs in the number of listed indications^a^

		Number of listed indications						
Substance / ingredient combination	Number of evaluated SmPCs	Arithmetic mean	Median	Min	Max	Range	SD	Absolute and relative frequencies of divergent SmPCs
Amoxicillin	10	13.7	14	9	16	7	1.8	2 (20%)
Ibuprofen	21	5.6	6	2	7	5	1.2	4 (19%)
Allopurinol	15	6.1	6	5	9	4	0.8	2 (13.3 %)
Clopidogrel	42	6.7	7	3	7	4	0.9	8 (19%)
Trimethorpim / sulfamethoxazole	8	9.4	9.5	7	11	4	1.7	5 (62.5%)
Escitalopram	20	4.8	5	1	5	4	0.9	1 (5%)
Ramipril	19	4.8	5	1	5	4	0.9	1 (5.3%)
Sertraline	25	4.7	5	1	5	4	1.1	2 (8%)
Acetyl salicilic acid	12	4.5	5	2	5	3	1.2	2 (16.7%)
Amoxicillin / clavulanic acid	21	8.1	8	8	11	3	0.7	1 (4.8%)
Omeprazole	27	9.7	10	7	10	3	0.8	3 (11.1%)
Amitriptyline	4	4.5	5	3	5	2	1.0	1 (25%)
Betamethasone	8	1.8	1	1	3	2	1.0	3 (37.5%)
Bisoprolol	19	2.3	2	1	3	2	0.7	9 (47.4%)
Esomeprazole	7	4.7	5	3	5	2	0.8	1 (14.3%)
Formoterol	8	2.8	2.5	2	4	2	0.9	4 (50%)
Levodopa / benserazide	7	2.1	2	1	3	2	0.7	3 (42.9%)
Metoclopramide	7	2.4	3	1	3	2	1.0	2 (28.6%)
Prednisolone	9	52.3	53	51	53	2	1.0	3 (33.3%)
Risperidone	28	3.7	4	2	4	2	0.7	4 (14.3%)
Torasemide	13	1.3	1	1	3	2	0.8	2 (15.4%)
Venlafaxine	29	4.9	5	3	5	2	0.5	2 (6.9%)
Budesonide	5	1.8	2	1	2	1	0.4	1 (20%)
Candesartan	24	3.0	3	2	3	1	0.2	1 (4.2%)
Cefaclor	7	4.9	5	4	5	1	0.4	1 (14.3%)
Ciprofloxacin	17	19.8	20	19	20	1	0.4	4 (23.5%)
Citalopram	25	1.6	2	1	2	1	0.5	9 (36%)
Codeine	3	1.3	1	1	2	1	0.6	1 (33.3%)
Ivy leaves	13	1.7	2	1	2	1	0.5	4 (30.8%)
Hydrochlorothiazide	18	4.6	5	4	5	1	0.5	7 (38.9%)
Lisinopril	19	3.9	4	3	4	1	0.3	2 (10.5%)
Lorazepam	6	2.8	3	2	3	1	0.4	1 (16.7%)
Macrogol	13	1.5	1	1	2	1	0.5	6 (46.2%)
Metoprolol	18	7.9	8	7	8	1	0.3	2 (11.1%)
Mometasone	6	1.5	1.5	1	2	1	0.5	3 (50%)
Pantoprazole	45	3.9	4	3	4	1	0.3	3 (6.7%)
Penicillin	9	6.9	7	6	7	1	0.3	1 (11%)
Pregabalin	32	3.0	3	2	3	1	0.3	3 (9.4%)
Salbutamol	10	1.8	2	1	2	1	0.4	2 (20%)
Salmeterol / Fluticason	12	1.8	2	1	2	1	0.4	2 (16.7%)
Valproic acid	15	2.5	3	2	3	1	0.5	7 (46.7%)
Amlodipine	27	3.0	3	3	3	0	0	0
Apixaban	4	3.0	3	3	3	0	0	0
Atorvastatin	25	2.0	2	2	2	0	0	0
Azithromycin	13	7.0	7	7	7	0	0	0
Candesartan / hydrochlorothiazide	21	1.0	1	1	1	0	0	0
Carvedilol	14	3.0	3	3	3	0	0	0
Cefuroxime	13	8.0	8	8	8	0	0	0
Cholecalciferol	4	1.0	1	1	1	0	0	0
Clindamycin	9	7.0	7	7	7	0	0	0
Dexamethasone / gentamicin	3	2.0	2	2	2	0	0	0
Diclofenac	6	6.0	6	6	6	0	0	0
Doxycycline	12	9.0	9	9	9	0	0	0
Enalapril	19	3.0	3	3	3	0	0	0
Enoxaparin	2	5.0	5	5	5	0	0	0
Estriol	4	1.0	1	1	1	0	0	0
Etoricoxib	21	5.0	5	5	5	0	0	0
Fentanyl	6	2.0	2	2	2	0	0	0
Formoterol / beclomethasone	4	2.0	2	2	2	0	0	0
Formoterol / budesonide	6	2.0	2	2	2	0	0	0
Fosfomycin	6	1.0	1	1	1	0	0	0
Furosemide	15	5.0	5	5	5	0	0	0
Gabapentin	27	3.0	3	3	3	0	0	0
Human insulin	5	1.0	1	1	1	0	0	0
Insulin aspart	5	1.0	1	1	1	0	0	0
Inuslin glargine	3	1.0	1	1	1	0	0	0
Inuslin lispro	3	1.0	1	1	1	0	0	0
Latanoprost	18	4.0	4	4	4	0	0	0
Lercanidipine	9	1.0	1	1	1	0	0	0
Levetiracetam	25	4.0	4	4	4	0	0	0
Melperone	9	3.0	3	3	3	0	0	0
Metamizole	12	5.0	5	5	5	0	0	0
Metformin	26	1.0	1	1	1	0	0	0
Metformin / sitagliptin	2	4.0	4	4	4	0	0	0
Methocarbamol	8	1.0	1	1	1	0	0	0
Methylphenidate	6	1.0	1	1	1	0	0	0
Mirtazapin	20	1.0	1	1	1	0	0	0
Mometasone	8	2.0	2	2	2	0	0	0
Moxonidine	12	1.0	1	1	1	0	0	0
Nebivolol	12	2.0	2	2	2	0	0	0
Ofloxacin	6	1.0	1	1	1	0	0	0
Opipramol	15	2.0	2	2	2	0	0	0
Oxycodone	28	1.0	1	1	1	0	0	0
Paracetamol	3	2.0	2	2	2	0	0	0
Phenprocoumon	5	3.0	3	3	3	0	0	0
Quetiapin	21	2.0	2	2	2	0	0	0
Ramipril / hydrochlorothiazide	18	1.0	1	1	1	0	0	0
Rivaroxaban	8	3.0	3	3	3	0	0	0
Simvastatin	32	2.0	2	2	2	0	0	0
Sitagliptin	6	7.0	7	7	7	0	0	0
Spironolactone	11	2.0	2	2	2	0	0	0
Tamsulosine	28	1.0	1	1	1	0	0	0
Tilidine / naloxone	8	2.0	2	2	2	0	0	0
Tiotropiumbromide	3	1.0	1	1	1	0	0	0
Tramadole	20	1.0	1	1	1	0	0	0
Valsartan	23	3.0	3	3	3	0	0	0
Valsartan / hydrochlorothiazide	26	1.0	1	1	1	0	0	0
Xylometazoline	27	1.0	1	1	1	0	0	0
Zolpidem	20	1.0	1	1	1	0	0	0
Zopiclone	18	1.0	1	1	1	0	0	0

*max* maximum value, *min* minimum value, *SD* standard deviation, *SmPC* summary of product characteristics

^a^presented in descending order by the range

**Table 4:** Other formal and content-related discrepancies between same-substance SmPCs in the presentation of indications

Substance / ingredient combination	Number of evaluated SmPCs	Absolute and relative frequency of SmPCs with discrepancy of type A	Absolute and relative frequency of SmPCs with discrepancy of type B
Amoxicillin	10	0	1 (10%)
Levodopa / benzerazide	7	1 (14.3%)	0
Amoxicillin / clavulanic acid	21	0	1 (4.8%)
Sulfamethoxazole / trimethoprim	8	0	1 (12.5%)

*SmPC* summary of product characteristics

**Table 5:** Discrepancies between same-substance SmPCs in the number of listed contraindications^a^

		Number of listed contraindications						
Substance / ingredient combination	Number of evaluated SmPCs	Arithmetic mean	Median	Min	Max	Range	SD	Absolute and relative frequencies of divergent SmPCs
Lisinopril	19	7.5	7	5	16	11	2.7	7 (36.8%)
Hydrochlorothiazide	18	9.1	9	2	11	9	1.9	7 (38.9%)
Torasemide	13	7.0	7	5	13	8	2.8	8 (61.5%)
Carvedilol	14	14.1	15	10	17	7	2.8	10 (71.4%)
Betamethasone	8	11.1	10.5	9	15	6	1.9	5 (62.5%)
Formoterol	8	2.3	2	1	7	6	2.0	4 (50%)
Lorazepam	6	5.8	7.5	2	8	6	3.0	3 (50%)
Metoprolol	18	15.0	16	10	16	6	1.5	8 (44.4%)
Oxycodone	28	6.8	7	6	12	6	1.1	13 (46.4%)
Mometasone	6	11	11	9	13	4	1.4	4 (66.7%)
Doxycyclin	12	4.5	5	3	6	3	1.1	6 (50%)
Estriol	4	8.5	8.5	7	10	3	1.7	2 (50%)
Lercanidipine	9	11.9	13	10	13	3	1.5	4 (44.4 %)
Metformin	26	7.0	7	6	9	3	1.0	14 (53.8%)
Metoclopramide	7	10.7	11	9	12	3	1.3	4 (57.4%)
Risperidone	28	1.2	1	1	4	3	0.6	4 (14.3%)
Spironolactone	11	9.2	9	8	11	3	1.0	4 (36.4%)
Tramadol	20	5.4	5	5	8	3	0.8	6 (30%)
Trimethoprim / sulfamethoxazole	8	9.9	10	8	11	3	1.0	4 (50%)
Xylometazoline	27	4.6	5	3	6	3	0.6	16 (59.3%)
Allopurinol	15	2.6	3	1	3	2	0.8	3 (20%)
Amlodipine	27	5.1	5	5	7	2	0.5	2 (7.4%)
Bisoprolol	19	12.5	12	12	14	2	0.6	9 (47.4%)
Cefaclor	7	4.4	4	4	6	2	0.8	2 (28.6%)
Cholecalciferol	4	7.3	7.5	6	8	2	1.0	2 (50%)
Codeine	3	10.3	11	9	11	2	1.2	1 (33.3%)
Enalapril	19	6.6	7	5	7	2	0.7	7 (36.8%)
Human insuline	5	1.8	1	1	3	2	1.1	2 (40%)
Metamizole	12	6.3	6	6	8	2	0.8	2 (16.7%)
Omeprazole	27	2.1	2	2	4	2	0.4	2 (7.4%)
Simvastatin	32	5.5	6	4	6	2	0.8	10 (31.3%)
Valproic acid	15	8.9	9	8	10	2	0.5	3 (20%)
Valsartan / hydrochlorothiazide	26	6.0	6	5	7	2	0.4	5 (19.2%)
Amitriptyline	4	7.3	7	7	8	1	0.5	1 (25%)
Atorvastatin	25	4.1	4	4	5	1	0.4	2 (8%)
Azitromycin	13	4.3	4	4	5	1	0.5	4 (30.8%)
Budesonide	5	1.8	2	1	2	1	0.5	1 (20%)
Candesartan	24	5.0	5	4	5	1	0.2	1 (4.2%)
Candesartan/hydrochlorothiazide	21	9.0	9	8	9	1	0.2	1 (4.8 %)
Cefuroxim	13	3.2	3	3	4	1	0.4	2 (15.4%)
Citalopram	25	5.1	5	5	6	1	0.3	3 (12%)
Clopidogrel	42	3.1	3	3	4	1	0.2	2 (4.8%)
Ivy leaves	13	1.8	2	1	2	1	0.4	2 (15.4%)
Formoterol/budesonide	6	1.8	2	1	2	1	0.4	1 (16.7%)
Fosfomycin	6	2.3	2	2	3	1	0.5	2 (33%)
Gabapentin	27	1.0	1	1	2	1	0.2	1 (3.7%)
Ibuprofen	21	10.6	11	10	11	1	0.5	9 (42.9%)
Levodopa / benserazide	7	12.1	12	12	13	1	0.4	1 (14.3%)
Melperone	9	5.7	6	5	6	1	0.5	3 (33%)
Methocarbamol	8	5.1	5	5	6	1	0.4	1 (12.5%)
Methylphenidate	6	9.2	9	9	10	1	0.4	1 (16.7%)
Mirtazapine	20	2.3	2	2	3	1	0.5	6 (30%)
Nebivolol	12	11.1	11	11	12	1	0.3	1 (8.3%)
Opipramol	15	9.7	10	9	10	1	0.5	4 (26.7%)
Pantoprazole	45	1.4	1	1	2	1	0.5	20 (44.4%)
Penicillin	9	3.6	4	3	4	1	0.5	4 (44.4%)
Ramipril	19	7.9	8	7	8	1	0.3	2 (10.5%)
Ramipril / hydrochlorothiazide	18	10.8	11	10	11	1	0.4	3 (16.7%)
Salbutamol	10	1.3	1	1	2	1	0.5	3 (30%)
Selmeterol / fluticasone	12	1.2	1	1	2	1	0.4	2 (16.7%)
Tiotropiumbromide	3	1.3	1	1	2	1	0.6	1 (33%)
Valsartan	23	4.1	4	4	5	1	0.3	2 (8.7%)
Venlaflaxine	29	3.3	3	3	4	1	0.5	9 (31%)
Zolpidem	20	5.4	5	5	6	1	0.5	7 (35%)
Zopiclone	18	6.3	6	6	7	1	0.5	6 (33.3%)
Acetyl salicilic acid	12	8.0	8	8	8	0	0	0
Amoxicillin	10	2.0	2	2	2	0	0	0
Amoxicillin / clavulanic acid	21	3.0	3	3	3	0	0	0
Apixaban	4	5.0	5	5	5	0	0	0
Ciprofloxacin	17	2.0	2	2	2	0	0	0
Clindamycin	9	2.0	2	2	2	0	0	0
Dexamethasone / gentamicin	3	7.0	7	7	7	0	0	0
Diclofenac	6	11.0	11	11	11	0	0	0
Enoxaparin	2	4.0	4	4	4	0	0	0
Escitalopram	20	5.0	5	5	5	0	0	0
Esomeprazole	7	3.0	3	3	3	0	0	0
Etoricoxib	21	11.0	11	11	11	0	0	0
Fentanyl	6	6.0	6	6	6	0	0	0
Formoterol/Beclomethason	4	1.0	1	1	1	0	0	0
Furosemide	15	8.0	8	8	8	0	0	0
Insulin aspart	5	1.0	1	1	1	0	0	0
Insulin lispro	3	2.0	2	2	2	0	0	0
Insulin glargine	3	1.0	1	1	1	0	0	0
Latanoprost	18	1.0	1	1	1	0	0	0
Levetiracetam	25	2.0	2	2	2	0	0	0
Macrogol	13	4.0	4	4	4	0	0	0
Metformin / sitagliptin	2	14.0	14	14	14	0	0	0
Mometasone	8	3.0	3	3	3	0	0	0
Moxonidine	12	5.0	5	5	5	0	0	0
Ofloxacin	6	2.0	2	2	2	0	0	0
Paracetamol	3	1.0	1	1	1	0	0	0
Phenprocoumon	5	8.0	8	8	8	0	0	0
Prednisolone	9	1.0	1	1	1	0	0	0
Pregabalin	32	1.0	1	1	1	0	0	0
Quetiapine	21	2.0	2	2	2	0	0	0
Rivaroxaban	8	6.0	6	6	6	0	0	0
Sertraline	25	3.0	3	3	3	0	0	0
Sitagliptin	6	1.0	1	1	1	0	0	0
Tamsulosin	28	3.0	3	3	3	0	0	0
Tilidine / naloxone	8	6.0	6	6	6	0	0	0

*max* maximum value, *min* minimum value, *SD* standard deviation, *SmPC* summary of product characteristics

^a^presented in descending order by the range

**Table 6:** Other formal and content-related discrepancies between same-substance SmPCs in the presentation of contraindications^a^

		Absolute and relative frequency of SmPCs with discrepancy of …				
Substance / ingredient combination	Number of evaluated SmPCs	Type A	Type B	Type C^b^	Type D	Combined type^b^
Metamizole	12	0	0	0	2 (16.7%)	0
Bisoprolol	19	0	1 (5.3%)	0	0	1 ^B+D^ (5.3%)
Metoprolol	18	0	0	5 ^1a^ (27.8%) 1 ^2b^ (5.6%)	0	0
Simvastatin	32	0	0	1^1b^ (3.1%). 6 ^2b^ (18.8%)	0	1 ^B+C2b^ (3.1%)
Torasemide	13	0	0	5 ^1a+b^ (38.5%)	1 (7.7%)	0
Metformin	26	1 (3.8%)	0	0	3 (11.5%)	3^B+D^ (11.5%)
Omeprazole	27	0	1 (3.7%)	0	0	0
Allopurinol	15	0	0	3 ^1b^ (20%)	0	0
Hydrochlorothiazide	18	0	0	1 ^2a+b^ (5.6%)	2 (11.1%)	0
Tramadole	20	0	0	0	5 (25%)	0
Levodopa / benserazide	7	0	0	0	1 (14.3%)	0
Phenprocoumon	5	2 (40%)	0	0	0	0
Zopiclone	18	0	0	0	4 (22.2%)	0
Metoclopramide	7	1 (14.3%)	0	0	0	0
Spironolactone	11	0	0	2 ^1.b^ (18.2%)	0	0
Valsartan / hydrochlorothiazide	26	1 (3.8%)	0	0	0	0
Lorazepam	6	0	0	2 ^1a+b^ (33.3%)	0	1 ^B+C1b^ (16.7%)
Amitriptyline	4	0	0	0	0	1 ^B+C2b^ (25%)
Doxycyclin	12	2 (16.7%)	0	4 ^1b^ (33.3%)	0	0
Estriol	4	0	0	0	0	2 ^B+C2b^ (50%)
Fosfomycin	6	1 (16.7%)	0	0	0	0
Penicillin	9	0	0	2 ^1a^ (22.2%)	0	0
Ivy leaves	13	5 (38.5%)	0	2 ^1b^ (15.4%)	0	0
Salmeterol / fluticasone	12	0	0	0	2 (16.7%)	0
Carvedilol	14	0	0	3 ^2b^ (21.4%)	0	7 ^B+C2b^ (50%)
Tiotropium bromide	3	0	0	0	1 (33.3%)	0
Betamethasone	8	3 (37.5%)	0	0	0	1 ^B+C1a^ (12.5%)
Trimethoprim / sulfamethoxazole	8	1 (12.5%)	2 (25%)	0	0	0
Codeine	3	2 (66.7%)	0	0	0	0
Mometasone	6	1 (16.7%)	1 (16.7%)	0	0	0
Formoterol	8	0	0	2 ^1a^ (25%)	0	0
Oxycodone	28	2 (7.1%)	0	0	0	0
Zolpidem	20	0	0	0	6 (30%)	0
Valproic acid	15	0	0	1 ^1b^ (6.7%)	0	0
Cefaclor	7	3 (42.9%)	0	0	0	0

*SmPC* summary of product characteristics

^a^presented in descending order by the number of annual prescriptions (exact figures are presented in Table 1)

^b^The superscript letters and numbers behind the absolute frequencies (e.g., ^1a^ or ^B+D^) indicate the respective subtype of discrepancy of type C or combination of two types of discrepancies; see Table 1 for definitions of types and subtypes of discrepancies

## Data Availability

The data evaluated in this study is available upon request. Please contact the corresponding author.

## Footnotes

1 In the AVR, the annual prescriptions referring to an active substance refer to the number of annual prescriptions of a drug with the respective active substance in any of the three available package sizes (N1, N2 or N3).

2 Based on the data obtained from "PharmNet.Bund", the dosage form with the most same-substance drugs was selected.

3 See: https://www.pharmnet-bund.de/static/de/index.html

4 From March 19, 2020 until August 30, 2020 the data of AMIS was migrated to a new database ("Arzneimittel-Antrags-Datenbank" [AmAnDa]) that features a new user interface ("Arzneimittel-Informationssystem certified edition" [AMIce]). During this period, AMIS data with the update status of March 19, 2020 were available. From August 31, 2020 on, AmAnDa was available and old AMIS data as of March 19, 2020 is still available. Therefore, during the period of data collection of this study (August 3, 2020 until October 27, 2020) we used AMIS data until August 30, 2020 and AmAnDa data from August 31, 2020 on. As a consequence, during August 3 until August 30, 2020 we obtained data with the update status of March 19, 2020.

5 The "Rote Liste" (see: https://rote-liste.de) is the German highest-circulation drug compendium (available online and print) which contains short information regarding human medicines approved in Germany (e.g., SmPCs and package inserts) and certain medical devices.

6 The "Gelbe Liste" (see: https://gelbe-liste.de) is a German drug compendium that is available online and print. It provides drug information as areas of application of a drug, contraindications, drug interactions, side effects, dosing, costs, SmPCs, etc.

7 Sixty out of 1486 identified products/SmPCs could not be obtained with either of the mentioned acquisition strategies.

8 For instance, in some same-substance SmPCs certain CInds are listed as separate points, whereas in other SmPCs relating to the same active substance these CInds are shown under one point. In these cases, the number of the listed CInds was evaluated as identical (= "No quantitative discrepancies in the number of Inds/CInds found"). Other, rather formal discrepancies, which were also not determined as discrepancies, referred to differing formulations (e.g. arterial hypertonia vs. hypertension). Furthermore, also the presence vs. absence of specifications of Inds/CInds (e.g. "concomitant use of drugs that are known to cause prolongation of the QT-interval" vs. "concomitant use of drugs that are known to cause prolongation of the QT-interval. Concomitant use of pimozide.") were not evaluated as discrepancies in the number of Inds/CInds. Finally, it has to be considered that CInds that refer to pregnancy and lactation are usually listed in a separate standardized paragraph (4.6 Fertility, pregnancy and lactation); however, in some SmPcs, CInds referring to pregnancy and lactation are (additionally) listed under point 4.2 (CInds) and in other SmPCs referring to the same active substance these CInds are listed only under point 4.6; this was evaluated as a discrepancy in the number of contraindications in the present study. However, such duplications of information in SmPCs were specifically evaluated and indicated in the secondary analysis.

9 Excluded active substances/combinations of active substances, reasons for exclusion, and ranking in the list of the most frequently prescribed active substances: levothyroxine sodium (ranking position 3), combination of levothyroxine and potassium iodate (ranking position 29) (both active substances were excluded as they are listed in the so called "Substitutionsausschlussliste"), imidazole/triazole combined with corticosteroids (ranking position 56), physiological saline solution (ranking position 76), noscapine (ranking position 90), ambroxol (ranking position 99), iron (II) glycine sulfate (ranking position 102) (the last five active substances were excluded as only one same-substance product was identified according to the study protocol).

10 This is an example for a group of included same-substance SmPCs with less than half harmonized SmPCs: Eight SmPCs referring to sulfamethoxazole/trimethoprim were evaluated. Three harmonized (= identical form and content) SmPCs were identified. The remaining five SmPCs featured a "unique" presentation of Inds.

## Code availability

All analyses were performed with Microsoft Excel (version 2019).
